# Barriers to research utilization and research use among registered nurses working in the care of older people: Does the BARRIERS Scale discriminate between research users and non-research users on perceptions of barriers?

**DOI:** 10.1186/1748-5908-3-24

**Published:** 2008-05-01

**Authors:** Anne-Marie Boström, Kerstin Nilsson Kajermo, Gun Nordström, Lars Wallin

**Affiliations:** 1 Department of Neurobiology, Care Sciences and Society, Division of Nursing, Karolinska Institutet, Stockholm, Sweden; 2 Department of Neurobiology, Care Sciences and Society, Division of Nursing, Karolinska Institutet and Clinical Research Utilization (CRU), Karolinska University Hospital, Stockholm, Sweden; 3 Department of Nursing, Karlstad University, Karlstad and Department of Neurobiology, Care Sciences and Society, Division of Nursing, Karolinska Institutet, Stockholm, Sweden

## Abstract

**Background:**

One strategy to enhance research use and change current practice is to identify barriers and then implement tailored interventions to reduce these barriers. In nursing, the BARRIERS scale has been frequently used to identify nurses' perceptions of barriers to research utilization. However, this scale has not been applied to care of older people, and only one study has investigated how identified barriers link to research utilization. Therefore, the purpose of this study was twofold: to describe RNs' perceptions of barriers to and facilitators of research utilization and to examine the validity of the BARRIERS scale in relation to research use.

**Methods:**

A cross-sectional survey design was used and registered nurses (RNs) working in the care of older people participated (response rate 67%, n = 140/210). Two questionnaires, the BARRIERS scale and the Research Utilization Questionnaire (RUQ), were used. Data were analyzed using descriptive and bivariate inferential statistics.

**Results:**

Characteristics of the organization and the presentation of research findings were rated as the most prominent barriers. The three items most frequently reported as barriers were: the nurse is isolated from knowledgeable colleagues with whom to discuss the research (89%); the facilities are inadequate for implementation (88%); and, the relevant literature is not compiled in one place (81%). Surveyed RNs suggested more support from unit managers and better availability of user-friendly reports in Swedish to enhance research use.

The RNs reported a modest use of research. A weak but significant correlation was found between the Research Use index in RUQ and the Presentation subscale in the BARRIERS scale (*r *= -0.289, *p *< 0.01), suggesting that the RNs reporting more research use were less likely to perceive presentation of research as a barrier. Dividing the sample into research users (n = 29) and non-research users (n = 105), the research users rated significantly lower on the subscales Presentation, Nurse and Research in the BARRIERS scale.

**Conclusion:**

The BARRIERS scale revealed differences in the perception of barriers between research users and non-research users. Thus, methodologically the scale appears useful in identifying some types of barriers to research utilization but not organizational barriers. The identified barriers, however, are general and wide-ranging, making it difficult to design useful specific interventions.

## Background

Today, old age is not an obstacle for undergoing advanced medical and surgical treatment for several health problems or medical diagnoses. For example, in Sweden about 2,500 persons older than 85 years underwent hip- or knee-joint operations in 2002 [1]. Increased knowledge in geriatrics and gerontological nursing and use of this knowledge contribute to better health for older people [1]. However, many studies have shown that a gap exists between what is known and what is done in practice, *i.e.*, many routines are still present in health care although research-based knowledge on more effective interventions are available [2]. One assumption to account for this persistent gap is that professionals in healthcare face diverse types of barriers that hamper them in changing clinical practice. Therefore, the main purpose of this study was to examine the validity of the BARRIERS scale in relation to research use, *i.e.*, assess the BARRIERS scale's capacity to discriminate perceptions of barriers between research users and non-research users.

### Perceptions of barriers to change and research utilization

To bridge the gap between 'the known and the done' a commonly suggested strategy is to identify barriers for changing practice and then implement interventions to reduce identified barriers. In a Cochrane review, Shaw and colleagues [3] found 15 randomized controlled trials using this strategy. There were various approaches for identifying barriers: investigators had predominantly used interviews or focus groups with professionals, but some used surveys among professionals or focus groups with patients. Because identified barriers varied widely, interventions were tailored to both the individual and the organization. For the most part, intervention arms of the included trials had a better outcome, though a meta-analysis of six trials was not statistically significant. Findings similar to this review were recently reported in a multiple-case analysis by Bosch and co-workers [4]. The authors selected 20 quality improvement studies reporting barriers analyses and tailored educational and organizational interventions. Various methods were used to identify barriers, and many investigators had combined several methods. The identified barriers were categorized into five types of barriers related to: patients, professionals, teams/social interactions, organizations, and structures. Few of the included studies, however, used a consistent approach to link the improvement intervention to identified barriers. Accordingly, there was a mismatch between barriers and selected interventions. To summarize, some evidence supports the strategy to change practice by using tailored interventions to overcome barriers. Yet, little is known about which barriers are valid, how these barriers should be identified, and what interventions are effective for overcoming barriers.

For more than 15 years, researchers have assessed nurses' perceptions of barriers to research utilization in which the BARRIERS scale has frequently been used [5-7]. Most of the studies have been conducted in North America and United Kingdom (UK). This instrument is linked to the Diffusion of Innovations theory [8]. The items in the instrument cover four domains of barriers: the nurse, the setting, the research, and the presentation. In a review including 39 published articles and six theses using the BARRIERS scale, nurses reported that the setting and the presentation domains contained the most prominent barriers [5]. Lack of time to read research reports and implement research in practice, lack of authority to change practice, lack of adequate facilities for implementation and lack of knowledge to interpret statistical analyses were reported as the most prominent obstacles. In many of the included studies the relationships between demographic data (*e.g.*, age, education, and professional experience) and barriers to research use were examined, but no clear patterns of relationships could be detected [5]. The studies were performed mainly among nurses in acute care settings (hospitals). Some of the studies were performed in primary care settings and in countries in Europe in addition to the UK. Even if minor differences were found, the findings were highly consistent across geographic location, time, setting, specialties, and groups of nurses.

One underlying assumption of the BARRIERS scale is that if barriers are reduced or eliminated, nurses' use of research will increase [6]. Only one study has used the BARRIERS scale to identify barriers and simultaneously measure nurses' use of research findings [9]. In that study, performed in a pediatric teaching hospital, one significant correlation was found between reported use of research findings and the nurse subscale, indicating that nurses who reported more research use were less likely to recognize characteristics related to the individual nurse as barriers than nurses who reported less use of research. If the BARRIERS scale is valid, then the scale should discriminate perceptions of barriers between nurses who report research use and those who do not. Are the barriers identified by this instrument related to nurses' research use, or are there other barriers that should be identified? The lack of support for relationships between perceived barriers and research use raises questions about the validity of the scale. In the UK, the content and construct validity of the scale has been questioned [10].

### The registered nurses' role in the care of older people in Sweden

In Sweden, the care of older people has shifted from hospitals to nursing homes and home care. The municipalities are responsible for the care of older people in accordance to the Swedish Health and Medical Care Act [11]. The majority (88%) of staff are enrolled nurses (ENs) and nurse aides (NAs) while the remaining 12% are registered nurses (RNs) and rehabilitation professionals (RPs), such as physiotherapists and occupational therapists [12]. RNs' work situation in the care of older people differs compared with RNs working in hospitals. In the care of older people RNs have a supervising role, performed through visiting the clients/patients in their home, making assessments, planning care, and evaluating provided care. The RNs instruct ENs and NAs on how to carry out the planned care of the clients/patients [13]. This role requires high medical, nursing, and pedagogical competence, as well as personal life experience [14]. In a Swedish study, RNs working in the care of older people expressed discontent with their work situation because of lack of time, lack of stimulation, and lack of support from managers. Overall, they emphasized the importance of a supportive organization [15].

In a previous study, we investigated staff use of research in the care of older people. Even if the staff reported positive attitudes to research, they reported a rather low use of research findings [16]. To enhance research use, and in that way enable evidence-based practice, the strategy of identifying barriers might be useful in developing adequate interventions. There is a lack of knowledge regarding to what extent RNs working in the care of older people perceive barriers to research utilization. The review by Shaw and colleagues [3], the study by Bosch and co-workers [4], and the two reviews on the BARRIERS scale [5,7] did not include any study from older people care settings. From a methodological point of view, there is a need to investigate whether the barriers identified by the BARRIERS scale are valid in relation to research use. The purpose of this study was therefore twofold: to describe RNs' perceptions of barriers to, and facilitators of, research utilization and to examine the validity of the BARRIERS scale in relation to research use, *i.e.*, the capacity of the Scale to discriminate perceptions of barriers between research users and non-research users.

## Methods

### Design

A cross-sectional survey design was used. The survey was carried out in 2001. The study was conducted after approval from the Research Ethical Committee at Huddinge University Hospital, Huddinge, Sweden (289/2000).

### Setting and participants

In Sweden, the south region of Stockholm consists of 10 municipalities representing about 500,000 inhabitants. In each of these municipalities, the Community Chief Nurse (CCN) was asked to participate, with eight expressing interest to be involved in the study. Two CCNs declined to participate because of other ongoing surveys among staff. Thus, from eight municipalities all RNs (n = 210) working in the care of older people were invited to participate. Two questionnaires were sent to the RNs with a cover letter outlining the purpose of the study, including assurance of confidentiality, voluntary participation, and the right to withdraw from participation at any time. Reminders were sent twice to those who did not respond. The response rate was 67% (n = 140/210).

### Data collection

Two questionnaires, the BARRIERS scale and the Research Utilization Questionnaire, were used to collect data.

### The BARRIERS scale

Funk and colleagues developed the BARRIERS scale from three separate sources: literature about research utilization, the Conduct and Utilization of Research in Nursing (CURN) project questionnaire, and informal data gathered from nurses [6]. The scale is composed of 29 items. The respondents were asked to rate to which extent they perceived each item as a barrier to the use of research findings. The respondents rated the items on a 4-point scale (1 = to no extent, 2 = to a little extent, 3 = to a moderate extent and 4 = to a great extent). In addition, a 'no opinion' alternative was offered. In the original study, factor analyses were performed that resulted in a four-factor solution [6]. One item did not load on any of the four factors. The four factors, or subscales, were assumed by Funk and colleagues to be congruent with dimensions in Rogers' Diffusion of innovations theory [8]. The subscales were labeled in accordance with Rogers' theory:

• the characteristics of the adopter – the nurse's research values, skills and awareness – the Nurse subscale (eight items).

• the characteristics of the organization – setting barriers and limitations – the Setting subscale (eight items).

• the characteristics of the innovation – qualities of the research – the Research subscale (six items).

• the characteristics of the communication – presentation and accessibility of the research – the Presentation subscale (six items).

The outcome of each subscale was calculated by adding each respondent's score and then dividing by the number of items in the subscale. The 'no opinion' responses were not used in calculating the outcome. In addition, in an open-ended question the respondents were asked to make suggestions on how to facilitate research utilization.

The BARRIERS scale has been translated into Swedish and then back-translated into English to confirm concordance [17]. An additional item was included covering the English language as a barrier for Swedish nurses. (This language item is not included in the subscales.) All items used in this present survey are presented in Table 1. Nilsson-Kajermo and colleagues used the BARRIERS scale in a sample of Swedish RNs working in hospitals and Cronbach's alpha was used to test the reliability of the scale [17]. The alpha values were 0.81 (the Nurse), 0.87 (the Setting), 0.86 (the Research) and 0.83 (the Presentation). In the present study the alpha values were 0.75 (the Nurse), 0.70 (the Setting), 0.78 (the Research) and 0.67 (the Presentation).

**Table 1:** Registered nurses reported barriers to research utilization (percentage of registered nurses' scoring 3 or 4 on the BARRIERS scale).

Subscale/item	Rank order	Total (n = 140)
**Nurse **(mean and SD)		2.19 ± 0.56
The nurse is isolated from knowledgeable colleagues with whom to discuss the research (n = 123)	1	89%
There is not a documented need to change practice (n = 96)	17	41%
The nurse does not feel capable of evaluating the research (n = 114)	19	39%
The nurse sees little benefit for self(n = 120)	21	33%
The nurse does not see the value of research for practice (n = 119)	22	30%
The nurse feels the benefits of changing practice will be minimal (n = 91)	24	28%
The nurse is unaware of the research (n = 132)	25	25%
The nurse is unwilling to change/try new ideas (n = 135)	28	19%
**Setting **(mean and SD)		2.71 ± 0.52
The facilities are inadequate for implementation (n = 124)	2	88%
The nurse does not have time to read research (n = 131)	5	79%
There is insufficient time on the job to implement new ideas (n = 127)	6	70%
Other staff are not supportive of implementation (n = 81)	9	63%
The nurse does not feel she/he has enough authority to change patient care procedures (n = 124)	13	50%
Physicians will not cooperate with implementation (n = 61)	14	46%
The nurse feels results are not generalizable to own setting (n = 113)	16	41%
Administration will not allow implementation (n = 70)	27	23%
**Research **(mean and SD)		2.17 ± 0.66
The research has not been replicated (n = 56)	10	57%
Research reports/articles are not published fast enough (n = 50)	12	52%
The literature reports conflicting results (n = 59)	20	37%
The nurse is uncertain whether to believe the results of the research (n = 108)	23	30%
The research has methodological inadequacies (n = 56)	26	23%
The conclusions drawn from the research are not justified (n = 81)	30	13%
**Presentation **(mean and SD)		2.62 ± 0.58
The relevant literature is not compiled in one place (n = 112)	3	81%
Research reports/articles are not readily available (n = 133)	4	80%
Implications for practice are not made clear (n = 121)	7	67%
The statistical analyses are not understandable (n = 125)	11	55%
The research is not reported clearly and readably (n = 90)	15	43%
The research is not relevant to the nurse's practice (n = 131)	29	17%
**No subscale/extra items**		
Research reports/articles are written in English (n = 130)	8	64%
The amount of research information is overwhelming (n = 93)	18	40%

### The research utilization questionnaire

To collect data on research use the Research Utilization Questionnaire (RUQ) was used. The RUQ was developed by Champion and Leach [18] and has been used in several studies [19]. The instrument has been described in detail elsewhere [16]. Research use is measured by a Research utilization in daily practice index (the RU index) consisting of nine items. The instrument employs a 5-point Likert scale (1 = strongly disagree, 2 = disagree, 3 = do not know, 4 = agree and 5 = strongly agree). After reversing the values of the negative statements, the index is calculated by adding each respondent's scores and dividing the sum by the number of items in the index.

The RUQ has been translated into Swedish and slightly revised [20]. Also in this case back-translation to English was used. Cronbach's alpha value for the RU index was 0.84 [20]. In the present study, the alpha value was also 0.84.

Additional questions were used to collect background data on age, education level (*i.e.*, nursing program and specialist nursing program) and work place.

### Data analysis

The data were analyzed using SPSS version 15 (Chicago, IL, USA). Descriptive analyses were used to present frequencies and distributions of reported barriers. To describe the RNs perceptions of barriers, the 4-point scale was dichotomized by merging the respondents who answered the two response alternatives 3 and 4 into one category representing respondents who reported the item as a barrier to research utilization. The respondents who scored response alternatives 1 and 2 were merged into a second expressing no perception of the item as a barrier. To analyze differences between ratings on the four subscales and the subgroups within the sample, the following background variables were dichotomized: nursing program (university level versus no university level), and workplace (nursing home versus specialist units, such as dementia group dwellings and rehabilitation).

To compare respondents who used research in clinical practice with respondents who did not the sample was divided into two groups. The ratings on the RU index were employed to evaluate respondents' use of research findings and an arbitrary cut-off value was set at 3.6, which represents 'research use behavior' more on the 'user-side' than on the 'do not know' or 'non-user-side' of the scale. The index consists of nine items and when a respondent rates, for example, *agree *(= 4) on five of the nine items and *do not know *(= 3) on four of the items, the mean value on the RU index will be 3.6. However, the data from six respondents could not be used because missing data for >50% of the items within the RU index. Thus, the sample consisted of 134 respondents for the analyses regarding the validity of the BARRIERS scale.

Pearson's product moment correlation coefficient was used to examine relationships between the four subscales, the RU index and the background variable age. Student's t-test was applied to assess differences between the means of the groups. A P-value <0.05 was considered to indicate statistical significance. On the open-ended question about factors facilitating research use, two authors (AMB and KNK) used the characteristics of the four subscales to categorize the respondents' answers.

## Results

### Description of the sample

The mean age of the RNs was 45 years (SD ± 9, range 23 to 62) and the majority (94%) was women. Of the 140 RNs, 44% had completed a nursing program at university level, *i.e.*, educated after 1982, which means that research methodology and nursing science were included in the curricula. Fifty-five (39%) had a specialist nursing program. The mean duration of the RNs working experience was 16 years (SD ± 10, range 0.5 to 40) and 60% of the RNs worked full-time. Seventy-three (52%) worked in nursing homes, 16 (11%) in rehabilitation units and the remaining (n = 51, 37%) worked in specialists units, such as a group dwelling for dementia and special houses.

### Barriers to and facilitators of research use in geriatric care

The most prominent barriers were found in the subscales Setting (mean = 2.71) and Presentation (mean = 2.62) (Table 1). The five barriers most highly rated were as follows: The nurse is isolated from knowledgeable colleagues with whom to discuss the research (89%), the facilities are inadequate for implementation (88%), the relevant literature is not compiled in one place (81%), research reports/articles are not readily available (80%), and the nurse does not have time to read research (79%) (Table 1).

Analyzing relationships between the outcomes of the four subscales and the background variables revealed that the RNs having an older nursing program before 1982 (and being older by age) without research methodology and nursing science in the curricula rated more barriers on the Presentation and Nurse subscales than the RNs having a recent nursing program (Table 2). RNs working in specialist units rated lower barriers on the Presentation subscale, as compared with RNs working in nursing homes.

**Table 2:** Results of the BARRIERS subscales in relation to background data.

		*Nurse*	*r*- or *t-*value	*P*-value	*Setting*	*r*- or *t-*value	*P*-value	*Research*	*r*- or *t-*value	*P-*value	*Presentation*	*r*- or *t- *value	*P*-value
Age^1^			0.233	<0.01		0.074	0.39		0.113	0.22		0.267	<0.01
Nursing program^2^	University	2.08			2.67			2.06			2.47		
		± 0.51			± 0.44			± 0.59			± 0.50		
	No university	2.31			2.77			2.15			2.76		
		± 0.59	2.195	0.03	± 0.55	1.040	0.30	± 0.71	0.680	0.50	± 0.67	2.632	0.01
Work place^2^	Nursing home	2.21			2.73			2.25			2.75		
		± 0.57			± 0.54			± 0.70			± 0.61		
	Specialist unit	2.17			2.68			2.09			2.47		
		± 0.55	-0.372	0.71	± 0.49	-0.607	0.54	± 0.60	-1.378	0.17	± 0.51	-2.892	<0.01

^1 ^Pearson's product-moment correlation coefficient.

^2 ^Students *t*-test

Sixty (43%) of the 140 RNs reported one or more suggestions that could facilitate research utilization. The most frequently suggested facilitators concerned setting (n = 58) and presentation (n = 48). Regarding the setting, respondents wanted support from unit managers, colleagues, and practice developers, as well as additional time for reading, discussing, and implementing research in practice. The RNs' proposals regarding presentation related to better accessibility of research findings. For example, research reports should be user-friendly, written in Swedish, and located close to the person's workplace. Some respondents suggested enhanced collaboration and establishment of networks. A few suggestions concerned educational activities.

### Research use and differences in perceptions of barriers

The mean score for the RU index was 2.95 (SD ± 0.80), indicating a modest degree of research use. A significant negative correlation was found between the respondents scoring on the RU index and the Presentation subscale, suggesting that the RNs reporting more use of research findings were less likely to perceive presentation of research as a barrier to research utilization (Table 3).

**Table 3:** Correlations between BARRIERS subscales and self-reported research use and comparisons between research users (RU) and non-research users (non-RU) reported barriers (subscales).

	Correlations^1^ Research use index vs. each subscale	*P-value*	RU n = 29^2^	Non- RU n = 105^2^	*t-value*^3^	*P-value*
Nurse subscale	-0.107	0.22	1.97 ± 0.53	2.25 ± 0.54	2.512	0.013
Setting subscale	-0.051	0.56	2.65 ± 0.45	2.71 ± 0.52	0.568	0.571
Research subscale	-0.171	0.06	1.96 ± 0.56	2.25 ± 0.66	2.139	0.035
Presentation subscale	-0.289	<0.01	2.36 ± 0.41	2.69 ± 0.61	3.422	0.001

^1 ^Pearson's product-moment correlation coefficient

^2 ^Loss of six respondents on the RU-index, n= 134

^3 ^Students *t*-test

To examine the validity of the BARRIERS scale in relation to research use, the sample was divided into two groups, the research users group (n = 29, 22%) and the non-research users group (n = 105, 78%). The research users group rated significantly lower (*i.e.*, less barriers) on the three subscales Presentation, Nurse and Research (Table 3). The lack of difference between the research users and non-research users groups on the Setting subscale led us to further examination on item level. No consistent trend was found regarding the two groups' ratings on items in this subscale (Table 4).

**Table 4:** The research users (RUs) and non-research users (non-RUs) reported barriers to research utilization regarding the Setting (*i.e.*, scoring 3 or 4 on the BARRIERS scale).

Items	RU n = 29^1^	Non-RU n = 105^1^
The facilities are inadequate for implementation (n = 124)	79%	90%
Other staff are not supportive of implementation (n = 81)	74%	57%
The nurse does not have time to read research (n = 131)	72%	82%
There is insufficient time on the job to implement new ideas (n = 127)	72%	70%
Physicians will not cooperate with implementation (n = 61)	56%	41%
The nurse does not feel she/he has enough authority to change patient care procedures (n = 124)	52%	48%
Administration will not allow implementation (n = 70)	23%	22%
The nurse feels results are not generalizable to own setting (n = 113)	21%	49%

^1 ^Loss of six respondents regarding the RU-index, thus the sample consists of 134 respondents

## Discussion

The RNs working in the care of older people reported barriers related to the Setting and the Presentation subscales. The RNs proposed better availability of research reports in Swedish, additional time for research use and support from unit managers for enhancing research utilization. The research users among the RNs rated significantly less barriers on the three Nurse, Research, and Presentation subscales than the non-research users. In the following, we will discuss reported perceptions of barriers and the potential usefulness of the BARRIERS scale for identifying barriers to research utilization.

### RNs' perceptions of barriers to and facilitators of research utilization

In general, the RNs working in the care of older people reported a rank order of barriers consistent with studies using the BARRIERS scale [7]. However, more than 75% of the RNs in the present study rated five of the 30 potential barriers as actual barriers (Table 1). To compare with another Swedish study with RNs working at a university hospital, only two items were rated as actual barriers by more than 75% of the RNs [21]. Thus, it appears as RNs working in the care of older people face more barriers than RNs working in hospitals. One reason might be that the average age of RNs working in the care of older people in Sweden is higher in comparison with RNs working in hospitals [22], implying that a greater proportion of nurses working in the care of older people have an older nursing program and lack courses in research methods and nursing science. In the present study, the RNs with an older nursing program rated barriers on the Presentation and Nurse subscales significantly higher than the RNs having a more recent nursing program at the university level. These comparisons suggest that many of the RNs working in the care of older people do not have sufficient knowledge that facilitates research use.

Nearly all RNs working in the care of older people reported lack of knowledgeable colleagues and inadequate facilities for implementation as the major barriers to research utilization. The RNs suggested establishment of networks among colleagues, staff, researchers, and physicians for promoting research use. In previous studies, lack of knowledgeable colleagues is not common among the top ten barriers [5,7]. In the care of older people in Sweden, the settings are mostly small units (such as nursing homes and group dwellings) with the intention to be 'homelike' for the residents. The majority of nursing staff are ENs and NAs and in many smaller units there is only one RN employed. In Sweden, ENs and NAs have nursing training within the upper secondary school, which can be compared with RNs who since 1982 have a nursing program at university level. Recent national surveys have shown that nearly half of ENs and NAs working in the care of older people do not have adequate training [22]. Moreover, smaller units have limited material and human resources for supporting practice development. These specific conditions in the care of older people are probably contributing to why so many RNs experience a shortage of knowledgeable colleagues. This finding is in accordance with a study among rural nurses who reported isolation and lack of a nursing research consultant as barriers to research utilization [23]. These results point to specific barriers of a situational and geographical nature. The RNs working in specialist units in the present study perceived fewer barriers on the presentation and accessibility of research (the Presentation subscale) than RNs working in nursing homes. Other studies have shown that, in comparison with working in general settings, working in specialist settings enhance research use [24].

The RNs' reports on lack of knowledgeable colleagues with who to discuss research may also be related to the English language. Two thirds of the RNs reported that the English language was a barrier to research use. In another Swedish study, almost half of the RNs working at a university hospital reported the English language and lack of knowledgeable colleagues as barriers [21]. This 'second language' barrier has also been identified as a major barrier among RNs in other non-English-speaking countries (*e.g.*, Norway, Finland and Greece) [25-27]. Generally, RNs prefer to use colleagues as information sources on research findings [28,29] and the need to have knowledgeable colleagues probably increases when the information is not in their own language.

Regarding the characteristics of the organization, many RNs reported lack of adequate facilities, lack of time to read and implement new ideas, and lack of support from other staff members as major barriers. These barriers have often been reported in previous BARRIERS studies [5,7]. Support from unit managers was one of the most frequently suggested factors to enhance the RNs' research use in the present study. Yet, respondents could not report lack of support from unit managers as a barrier because there is no item in the BARRIERS scale explicitly measuring the perception of support from front-line managers. One item is formulated 'Administration will not allow implementation', which, in the present study, was ranked as the 27^th ^barrier of 30. We interpret this finding to mean this item does not measure the concept of 'leadership'. Administration is a concept that in a Swedish context and language refers to and represents something impersonal and higher up in the organization. The wording 'not allow' also seems to be inappropriate to use in the care of older people. Such a setting often consists of small units and few staff categories, all of which implies a less formal organization. We consider it important to extend the BARRIERS scale with an item that measures support from front-line managers because the relationship between research use and leadership is well documented [24,30]. One positive finding was that in the present study the RNs did not report lack of authority to change practice as a major barrier. This finding is in contrast with what has been found in other studies [5]. One of the advantages with working in smaller settings with less hierarchy might be that the RNs have more authority in influencing and putting evidence into practice.

### Differences between reported research use and perceived barriers

A significant negative correlation was found between the RU index and the Presentation subscale, demonstrating that the RNs working in the care of older people that scored more use of research findings rated lower barriers related to the presentation and accessibility of research. These results differ from the findings of McCleary and Brown, who reported a significant correlation between research use and the Nurse subscale among nurses working in a pediatric teaching hospital [9]. It is logical that if RNs do not have access to or do not understand the presentation of research findings in research reports, they will not use research findings in practice. Being aware of relevant research is the first stage in implementing findings according to Rogers' Innovation-Decision process [8]. Access to research findings at the work place has also been identified as a determinant of research uptake in the care of older people [31]. The statistical analyses demonstrated that the scale detected certain differences between research users and non-research users. The research users group rated significantly lower on three of the four subscales as compared with the non-research users group, indicating that the RNs using research perceived fewer barriers than those not using research (Table 3). These results support the underlying assumption of the BARRIERS scale, *i.e.*, lower perceptions of barriers imply more research use and visa versa [6]. However, the lack of significant correlation between the RU index and the Setting subscale, and especially the lack of significant difference between the two groups' ratings on this subscale, is thought-provoking. The literature on barriers notoriously reports the Setting as the predominant barrier to research utilization [5,7]. Examining the two groups' ratings on the items in this subscale revealed that there was no consistent trend in the results (Table 4). The findings suggest that the Setting subscale measures heterogeneous characteristics of the organization, which appear to have different implications for research users compared with non-users. These differences are challenging to understand, especially when the goal is to identify adequate interventions for decreasing barriers to research use.

### Is the BARRIERS scale useful to identify barriers to research utilization?

The BARRIERS scale measures perceptions of barriers regarding the nurse, the setting, the research, and the presentation of research. These four types of barriers to research use are, to some extent, congruent with the five types of barriers presented by Bosch and co-workers [4], except from barriers related to patients and the wider environment (the structure), which are lacking in the BARRIERS scale. In the present study, the BARRIERS scale exposed differences in the perceptions of barriers to research utilization between research users and non-research users on the Nurse, the Research and the Presentation subscales, indicating that the instrument appears useful for identifying these types of barriers to research utilization. The lack of difference between the two groups on the predominantly reported Setting subscale undermines the validity of the BARRIERS scale to identify organizational barriers to research utilization. A further issue is that the instrument identifies barriers generally and with wide-ranging characteristics, making it difficult to design specific, tailored interventions to decrease the barriers. Previously, only one study has used the BARRIERS scale to identify barriers to actually promote research use in practice [32]. Based on a pre-survey, Fink and co-workers implemented multiple organizational interventions, which ranged from integration of evidence-based practice philosophy into nursing job descriptions to establish unit-based journal clubs. The multi-faceted intervention significantly decreased the nurses' ratings on the Setting subscale, but which of the components of the organizational intervention that made this reducing effect was not possible to distinguish. This difficulty in designing specific interventions to reduce barriers is not unique. Findings reported by Shaw and colleagues [3] and Bosch and co-workers [4] point to a lack of useful theory for tailoring interventions to address barriers.

### Methodological consideration

All RNs in participated municipalities were invited and a response rate of 67% was achieved, which must be judged as sufficient when using postal questionnaires [33]. The study was performed in eight municipalities of varying sizes. These municipalities hold about one-third of the RNs working in the care of older people in Stockholm County. We believe our sample is representative for an urban region in Sweden. Conditions, such as turnover and lack of required training among staff, can differ between the care of older people in city regions and rural regions [34] and our findings are probably not generalizable to all Swedish or international care of older people. The two questionnaires (BARRIERS scale and RUQ) have been used in several international and Swedish studies where they have been judged to be valid and reliable measures. In this study, the four subscales from the BARRIERS scale and the RU index from the RUQ were used. The validity of three of the BARRIERS subscales is supported by the current study. The reliability was tested using Cronbach's alpha statistics, and the measures were sufficiently consistent [33]. The respondents' answers on the open-ended question on factors to facilitate research use supported their scoring on the barriers subscales, *i.e.*, they reported supportive factors mainly in accordance with their reported type of barriers. Some of the items in the research subscale in the BARRIERS scale had high proportions of no opinion responses. This finding has been reported in many studies using the BARRIERS scale [5,7] and indicates a lack of validity of this subscale.

## Conclusion

A great proportion of RNs working in the care of older people perceived several barriers to research utilization. The barriers mainly concerned the characteristics of the organization and the presentation and accessibility of research findings. The RNs also reported the lack of knowledgeable colleagues as a major barrier. To enhance research use in the care of older people, strategies should focus on organizational issues, including supportive leadership by the unit managers and collaboration between colleagues, staff, and physicians. Furthermore, user-friendly guidelines in Swedish, with clear implications for practice, should be implemented. On a methodological base, the BARRIERS scale appears to be useful to identify some types of barriers to research utilization, but identified barriers are general and wide-ranging, making it difficult to design useful specific interventions. Future research should focus on identifying relevant organizational barriers and effective interventions to reduce identified barriers.

## Competing interests

The authors declare that they have no competing interests.

## Authors' contributions

AMB, KNK, GN and LW designed the study. AMB and KNK performed the data collection and analysis. AMB was the principal author of the manuscript though significant contributions were made from all co-authors. All authors have read and approved the final manuscript.
